# A Treatise on Therapeutics, and Pharmacology or Materia Medica

**Published:** 1856-12

**Authors:** 


ART. IV.—*A Treatise on Therapeutics, and Pharmacology or Materia Medica.* By GEORGE B. WOOD, M. D., late President of the American Medical Association, President of the College of Physicians and Surgeons, Professor of the Theory and Practice of Medicine in the University of Pennsylvania, senior Physician of the Pennsylvania Hospital, one of the Authors of the U. S. Dispensatory, author of a Treatise on the Practice of Medicine, etc., etc. In Two Volumes, 8vo. Philadelphia: I. B. LIPPINCOTT & Co. 1856.

Prof. Wood sends out these portly volumes as a parting gift of authorship to that profession which has so highly honored him, during a long, brilliant, and unspotted career as a medical man. The coming on of old age warns him that his day of labor approximates to a close, and though he will, in the natural course of events, toil on yet longer in unimpaired usefulness and honor for a time, he can hardly hope to accomplish another heavy labor of authorship.

In speaking of Wood's Practice of Medicine, we have not hesitated to criticise its too strong reliance on remedial agencies, and its propensities to too active treatment. But the very training and personal qualities which interfered with his full success, (in our opinion) as a writer on treatment, are those which do most amply fit him for a work like the one under consideration, and give to it additional value. He who for thirty years has studied medicines rather than disease, may over value their efficacy, but he is by far the more competent judge of their *comparative* merits. In all matters of therapeutical and pharmacological art, Prof. Wood has no superior.

The result is in these volumes, full of sound judgment and prudent instruction. We like the scheme of the book. It evinces the teacher, rather than the writer. The classification is regardless of botanical or chemical subdivisions or arrangements. Remedies are classified, not by the form of a flower, but by their therapeutic effects. And, in his list of remedies, we find some unknown to the apothecary, but on the skillful use of which many a physician must gain or lose a reputation. Thus, under the head of *tonics*, we have able articles on the therapeutic action of Diet, Exercise, Pure Air, Mental Influences, Traveling, Cold, and Transfusion of Blood. Few persons, however familiar with these most essential of remedies, can peruse these chapters without acquiring either new knowledge, or a better and more definite understanding of their former impressions.

But a full review is out of our power. Of course, in such a work, there is no dominant idea which the critic may seize and elaborate upon. Coming to us in a season of constant occupation, and accompanied by other works, also demanding attention, we are compelled to dismiss Wood's Therapeutics without that full treatment which its merits demand. But we cannot do so without a word or two of criticism upon one point. We recognize, everywhere, in the author's remarks upon the adaptation of remedies to disease, what we consider a too full reliance on depleting remedies, and a want of faith in tonics. We are, perhaps, an extremist on this point. Certainly, few would set our opinion against that of Prof. Wood, but observation of the effect of remedies, to some extent, and observation of the operations of nature to a still greater, have given us an abiding dislike to any, even momentary, lessening the powers of life by medicines, and a growing faith in the universality of the tonic treatment. Theoretically, we must consider even a depleting measure, so far as it conduces to health, as a tonic; and a tonic injudiciously administered, is equally a depletory measure; but we object that Prof. Wood sees more danger in tonics than in depletion, and bestows a wider importance upon the latter than the former.

In this, we doubt whether experience will bear him out, and we are sure that the present tendency of the medical mind is not sufficiently drawn away from the sanguinary ideas which originated in the loose pathology of past times. Doubtless, in the swinging of the pendulum we shall go beyond the central line of truth, and run as far toward support as we have heretofore gone in depletion; but the movement that-wise is yet slow, the danger has not yet arrived, and, in the meantime, the lives of our patients are exposed to the greater hazards of depletion. It is difficult to choose the middle path in this matter, but for one we feel as yet no danger from the progressive idea, and shall wait a season before acting the part of a conservative.

In this brief general criticism we have embodied all our objections to the work under notice. The fault (and many will consider it anything but a fault) is based upon a general principle, a cardinal point of faith in the author. Accordingly we find it running through the work, and giving tone to the discussion of individual remedies.

Otherwise, in all matters of physiological discussion, in the accurate and learned chemistry, in the carefully correct botanical descriptions, and in the estimates of the comparative value of remedies of any one class, the work is perfect. Written in a style of true eloquence; neat, but not ornate; never wearisome, and characterized by great fairness and honesty of opinion; full, without diffuseness, giving most space were space is most needed; leaving unmentioned many of the worthless articles with which our materia medica is incumbered, and cutting off others of the same sort with a paragraph; and, finally, dispensing almost altogether with formulæ of administration, of which he justly remarks that "They lead to an indolent reliance on mere authority, by sparing the trouble of thought; and greatly conduce to an empirical and routine practice, neither creditable to the physician, nor profitable to the patient," the work is one which we can truthfully commend as a valuable and permanent addition to American medical literature. To the practitioner, as a mere work of daily use for reference, it is, perhaps, the best within reach.

For sale by PHINNEY & Co.

